# Supplementary material for: HIVprotI: an integrated web based platform for prediction and design of HIV proteins inhibitors
Source: J Cheminform. 2018 Mar 9;10:12. doi: 10.1186/s13321-018-0266-y (PMC5845081; doi:10.1186/s13321-018-0266-y)
Supplement: Supplementary file 2 — Additional file 2. Source code of HIVProtI web server. [file 13321_2018_266_MOESM2_ESM.zip › HIVprotI_Source-code/hivprotI/weka-3-6-6/doc/weka/gui/graphvisualizer/BIFParser.html]

BIFParser


---


|  |  |  |
| --- | --- | --- |
| **Overview** \| **Package** \| **Class** \| **Tree** \| **Deprecated** \| **Index** \| **Help** \| **Weka's home** | |  |
| **PREV CLASS**   **NEXT CLASS** | **FRAMES**    **NO FRAMES**     **All Classes** |  |
| SUMMARY: NESTED \| FIELD \| CONSTR \| METHOD | DETAIL: FIELD \| CONSTR \| METHOD |  |


---


## weka.gui.graphvisualizer Class BIFParser

```
java.lang.Object
  weka.gui.graphvisualizer.BIFParser
```

**All Implemented Interfaces:**: GraphConstants

---

``` public class BIFParser extends java.lang.Object implements GraphConstants ```

This class parses an inputstream or a string in
XMLBIF ver. 0.3 format, and builds the datastructures
that are passed to it through the constructor.

**Version:**
:   $Revision: 7059 $ - 24 Apr 2003 - Initial version (Ashraf M. Kibriya)

**Author:**
:   Ashraf M. Kibriya (amk14@cs.waikato.ac.nz)

---

**Field Summary**

**Fields inherited from interface weka.gui.graphvisualizer.GraphConstants**

`DIRECTED, DOUBLE, NORMAL, PLURAL_DUMMY, REVERSED, SINGULAR_DUMMY`


**Constructor Summary**

`BIFParser(java.io.InputStream instream,
FastVector nodes,
FastVector edges)`
  
          Constructor (if our input is an InputStream)

`BIFParser(java.lang.String input,
FastVector nodes,
FastVector edges)`
  
          Constructor (if our input is a String)


| **Method Summary** | |
| --- | --- |
| `java.lang.String` | `parse()`             This method parses the string or the InputStream that we passed in through the constructor and builds up the m\_nodes and m\_edges vectors |
| `static void` | `writeXMLBIF03(java.lang.String filename, java.lang.String graphName, FastVector nodes, FastVector edges)`             This method writes a graph in XMLBIF ver. |

**Methods inherited from class java.lang.Object**

`equals, getClass, hashCode, notify, notifyAll, toString, wait, wait, wait`

**Constructor Detail**

### BIFParser

```
public BIFParser(java.lang.String input,
                 FastVector nodes,
                 FastVector edges)
```

:   Constructor (if our input is a String)

    **Parameters:**: `input` - the string to be parsed (should not be null): `nodes` - vector containing GraphNode objects (should be empty): `edges` - vector containing GraphEdge objects (should be empty)

---


### BIFParser

```
public BIFParser(java.io.InputStream instream,
                 FastVector nodes,
                 FastVector edges)
```

:   Constructor (if our input is an InputStream)

    **Parameters:**: `instream` - the InputStream to be parsed (should not be null): `nodes` - vector containing GraphNode objects (should be empty): `edges` - vector containing GraphEdge objects (should be empty)


**Method Detail**

### parse

```
public java.lang.String parse()
                       throws java.lang.Exception
```

:   This method parses the string or the InputStream that we
    passed in through the constructor and builds up the
    m\_nodes and m\_edges vectors

    :   **Returns:**: returns the name of the graph **Throws:**: `java.lang.Exception` - if both the inString and inStream are null, i.e. no input has been provided: `BIFFormatException` - if there is format of the input is not correct. The format should conform to XMLBIF version 0.3: `java.lang.NumberFormatException` - if there is an invalid char in the probability table of a node.

---


### writeXMLBIF03

```
public static void writeXMLBIF03(java.lang.String filename,
                                 java.lang.String graphName,
                                 FastVector nodes,
                                 FastVector edges)
```

:   This method writes a graph in XMLBIF ver. 0.3 format to a file.
    However, if is reloaded in GraphVisualizer we would need to layout
    the graph again to display it correctly.

    :   **Parameters:**: `filename` - The name of the file to write in. (will overwrite): `graphName` - The name of the graph. (will be the name of network tag in XMLBIF): `nodes` - Vector containing all the nodes: `edges` - Vector containing all the edges


---


|  |  |  |
| --- | --- | --- |
| **Overview** \| **Package** \| **Class** \| **Tree** \| **Deprecated** \| **Index** \| **Help** \| **Weka's home** | |  |
| **PREV CLASS**   **NEXT CLASS** | **FRAMES**    **NO FRAMES**     **All Classes** |  |
| SUMMARY: NESTED \| FIELD \| CONSTR \| METHOD | DETAIL: FIELD \| CONSTR \| METHOD |  |


---
